# Multisegmental spondylitis due to *Tropheryma whipplei*: Case report

**DOI:** 10.1186/1750-1172-4-13

**Published:** 2009-06-03

**Authors:** David Spoerl, Diego Bär, Julian Cooper, Thomas Vogt, Alan Tyndall, Ulrich A Walker

**Affiliations:** 1 Department of Rheumatology, University of Basel, Felix Platter Spital, Basel, Switzerland

## Abstract

We report a patient who presented with inflammatory back pain due to multisegmental spondylitis. Following a vertebral biopsy which failed to detect an infectious organism, the patient was treated with etanercept, a tumor necrosis factor (TNF)-α inhibitor, for suspected undifferentiated spondyloarthritis. The back pain worsened and the spondylitic lesions increased. Only in a vertebral rebiopsy with polymerase chain reaction (PCR) amplification of *Tropheryma whipplei*, the causative agent of Whipple's disease was identified. *Tropheryma whipplei *should be considered as a cause of spondylitis even with multisegmental involvement and in the absence of gastrointestinal symptoms. In this clinical setting, routine PCR for *Tropheryma whipplei *from vertebral biopsies is recommended.

## Background

Low back pain is commonly conceptualised and managed as being either mechanical or inflammatory in nature. The inflammatory origin of low back pain can be sensitively detected by magnetic resonance imaging (MRI) [1]. Frequently patients with spondylitis suffer from "autoimmune" spondyloarthritis, a group of disorders that includes ankylosing spondylitis, reactive arthritis, and the spondylitis that may accompany psoriasis and inflammatory bowel diseases. All these forms of spondylitis are amenable to treatment with TNF-α blockers. Infection should be ruled out in atypical cases by searching for foci, blood culture and eventually vertebral biopsy. In contrast to the multisegmental spinal involvement typical of the "autoimmune" spondyloarthritis group of disorders, it is unusual for infectious agents to manifest with spondylitis on multiple levels in the lumbar spine [2]. Staphylococcus aureus is the most common organism involved in spinal infections and is thought to hematogenously spread through the paravertebral collateral arteria into the vertebral bone marrow [3].

Whipple's disease is a rare, multisystemic infection caused by *Tropheryma whipplei*. Symptoms usually include diarrhea, weight loss, malabsorption and migratory oligo- or polyarthritis. The arthropathy usually precedes gastrointestinal symptoms by years. Diagnosis is generally made by small bowel biopsy or PCR amplification from the biopsy specimen [4].

We report the first case of multisegemental spondylitis due to infection with *Tropheryma whipplei*. Following a first negative result of a vertebral biopsy in which PCR amplification has not been performed, the patient was erroneously treated with a TNF-α inhibitor for suspected undifferentiated spondyloarthritis.

## Case presentation

**Figure 1:**
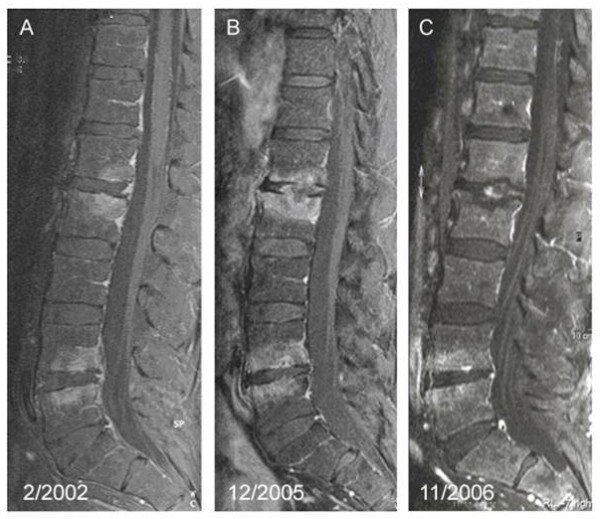
**MRI alterations during follow-up**. Contrast-enhanced, T1 weighted, fat saturated MRI demonstrating progression of two spondylitic lesions with the onset of clear erosions in the L1/L2 segment during treatment with etanercept, a TNF-α blocking agent between years 2002 and 2005. After cessation of the TNF-α blocker and initiation of antibiotic treatment, the multisegmental spondylitis regressed (year 2006).

In the year 2002, a 64 year old man presented with a 5 year history of inflammatory back pain and repeated, bone-scan confirmed, transient flares of arthritis involving one proximal interphalangeal, both tibiotalar, and tarsometarsal joints. His physical examination was otherwise normal. Laboratory tests revealed inflammation (erythrocyte sedimentation rate (ESR) 34 mm/h, C-reactive protein (CRP) 164 g/L). A contrast-enhanced, T1 weighted, fat saturated MRI scan of the spine showed contrast enhancing lesions in the first (L1) and second (L2) as well as fourth (L4) and fifth (L5) lumbar vertebra which spared the intervertebral discs (Fig 1A). Biopsy of the second lumbar vertebral (L2) lesion failed to show inflammation; cultures were negative. Chest X-ray, abdominal ultrasonography, transesophageal echocardiography, gastroduodenoscopy, and colonoscopy were normal, as were blood cultures and searches for mycobacteria and HIV. He was then started on low-dose corticosteroids and methotrexate had been added to the regimen in August 2004.

After disease progression, methotrexate had been replaced by anti-TNF-α treatment with etanercept in August 2005 for suspected undifferentiated spondyloarthritis. Consequently, lethargy, night-sweats, and weight loss (10 kg in 6 months) developed. No other gastrointestinal symptoms existed. The back pain and inflammation (ESR 60 mm/h and CRP 860 g/L) worsened. A control MRI (Fig 1B) showed erosive disease. A rebiopsy of the second lumbar vertebra was performed. PCR from the vertebral biopsy amplified a DNA product, which was confirmed by sequencing to originate from *Tropheryma whipplei*. Retrospectively, the same pathogen was detected by PCR in the gastric biopsy from the year 2002. No PCR amplification had been carried out in the cerebrospinal fluid. Coinfection with *Giardia lamblia *was diagnosed by repeat gastroduodenoscopy which at this time also revealed the typical periodic acid-Schiff (PAS)-positive macrophages in the duodenal mucosa.

The immunosuppressive treatment was stopped and sequential treatment of *Tropheryma whipplei *with ceftriaxon and cotrimoxazol was initiated in February 2006. *Giardia lamblia *was treated with metronidazol. After 12 months of treatment, the patient was in clinical remission with marked improvement of the spinal lesions (Fig 1C). In July 2007, cotrimoxazol was switched to doxycyclin plus hydroxychloroquine because the laboratory markers of inflammation were still mildly elevated (CRP 5.5 mg/l, ESR 26 mm/h). In August 2008 there were no clinical or serological signs of inflammation, another control MRI showed no signs of activity. Treatment was discontinued in October 2008 due to doxycyclin induced phototoxic skin eruptions [5]. At his last follow-up in February 2009, there was no sign of relapse.

## Conclusion

Spondylitis is a rare manifestation of Whipple's disease. Multisegmental involvement has to our knowledge not been reported before. The first symptom of Whipple's disease usually is a polyarthritis. Typically the diagnosis of the underlying infection is delayed for several years [6]. The physician and rheumatologist should be aware of this rare differential diagnosis of arthritis and spondyloarthritis. Erosive intervertebral disease and a L4/5 location of inflammatory lesions are not typical for primary spondyloarthritis. An accelerated progression and onset of new gastrointestinal symptoms has been described in a few cases of Whipple's disease treated with non-biologic and biologic immunosuppressants [7]. A septic, life-threatening disease course has been reported under immunosuppression [8]. *Tropheryma whipplei *should be considered even in the absence of gastrointestinal symptoms; therefore routine PCR from vertebral biopsies is recommended [9]. Typically, the diagnosis is made by PAS staining of small-bowel-biopsy specimens and occasionally also other tissues, which on light microscopy show magenta-stained inclusions within macrophages. Immunohistochemistry may provide greater sensitivity and specificity than does PAS staining but is not widely available. Our case suggests that PCR from gastrointestinal sources can be more sensitive than the demonstration of PAS-positive macrophages in histopathology. Last but not least, the case provides further support for an association between Whipple's disease and *Giardia lamblia *infection [10]. The pathogenesis of this coinfection is unclear but may be promoted by a common immune defect, a common source of infection, or the circumstance that infection with one organism may predispose to infection with the other [10].

If untreated, Whipple's disease is invariably fatal. An effective antibiotic regimen consists of ceftriaxone (2 g daily) for 2 weeks, followed by cotrimoxazole (160 mg of trimethoprim and 800 mg of sulfamethoxazole twice per day) for 1 to 2 years. In case of an unsatisfactory clinical response or a relapse, doxycycline (200 mg/day) plus hydroxychloroquine (200 mg three times per day), an alkalinizing agent which decreases the viability of *Tropheryma whipplei *in phagosomes, is recommended [10]. Typical histopathological findings in duodenal biopsies can however persist for several years after successful antibiotic therapy [11].

## Abbreviations

TNF: Tumor necrosis factor; PCR: Polymerase chain reaction; MRI: Magnetic resonance imaging; ESR: Erythrocyte sedimentation rate; CRP: C-reactive protein; HIV: Human immunodeficiency virus; DNA: Deoxyribonucleic acid; PAS: Periodic acid-Schiff.

## Consent

Written informed consent was obtained from the patient for publication of this case report and accompanying images. A copy of the written consent is available for review by the Editor-in-Chief of this journal.

## Competing interests

The authors declare that they have no competing interests.

## Authors' contributions

All authors treated the patient during the years 2002 to 2008. Each of them have made substantial contributions to data acquisition and interpretation. All have been involved in drafting the manuscript.

UAW critically revised the manuscript for important intellectual content and has given final approval of the version to be published. All authors read and approved the final manuscript.
